# Using coupons to encourage healthier child snack purchases in corner stores: results from the CHOMPS study

**DOI:** 10.3389/fnut.2024.1290710

**Published:** 2024-01-17

**Authors:** Megan E. Mayer, Anna R. McAlister, Christina D. Economos, Suzanne Mack, Kaela Plank, Sean B. Cash

**Affiliations:** ^1^Department of Nutrition and Health Studies, Framingham State University, Framingham, MA, United States; ^2^Gerrish School of Business, Endicott College, Beverly, MA, United States; ^3^Friedman School of Nutrition Science and Policy, Tufts University, Boston, MA, United States

**Keywords:** children, adolescents, corner stores, healthy snacks, nutrition incentives

## Abstract

**Objectives:**

To examine youths’ (ages 6–15 years) autonomous snack purchases in corner stores and pilot use of coupons to encourage more healthful snack purchases.

**Methods:**

This pilot study involved four corner stores proximal to K-8 schools in Massachusetts. Kids-only coupons of varying discounts were provided in store and paired with simple visual and verbal economic and health messages. Observational data about youths’ autonomous snack purchases was recorded pre- and post-intervention. Outcomes of interest were snack item, price, and nutrient content. Comparisons of purchase characteristics and nutritional content across intervention conditions were made using Chi-squared and *t*-tests.

**Results:**

Across all stores, 2,973 purchase observations were recorded totaling approximately $6,000. Researchers estimated that about 55% of shoppers were 10–12 years old. Modest coupon usage (2.2% of purchases) was noted. However, candy purchases decreased, and the percentage of purchase events that included at least one healthier food item more than doubled, regardless of coupon use. Improvements in the nutritional content of snacks were also observed.

**Conclusion:**

Kids-only coupons have the potential to assist with shifting autonomous snack purchase behavior in outside of school settings.

## Introduction

Starting as early as second and third grade, children in the U.S. spend considerable amounts of money on independent food purchases in corner and convenience stores, with much of this spent on energy-dense, nutrient-poor (EDNP) foods (1–9). Limited access to affordable, healthy options and the disproportionately high presence of convenience stores in lower income and/or minority communities, when compared to higher income, majority white communities, can influence child purchasing decisions, especially in neighborhoods where many children walk to and from school. In turn, children’s purchasing power and the neighborhood environment can be linked to health disparity concerns as children of lower socioeconomic or minority backgrounds are more likely to purchase EDNP foods (10–14). Veur et al. (10) found African American students were more likely to report corner store purchases than children of other races.

Observational studies report youth purchasing behaviors to be influenced by a multitude of factors including: peer influences at time of snack purchase, the landscape of the built environment surrounding a child’s home and school, and product pricing (15–22). Previous studies report that when children are spending alone, price reductions of as little as $0.10 can influence a child’s purchasing decisions (5), suggesting that incentives can be an effective way to promote healthier food items.

Experimental studies corroborate the influence of food cost on purchasing behavior in both adults and children. Within a simulated convenience store setting, taxing higher calorie food items in conjunction with subsidies for lower calorie food items was found to dis-incentivize adolescents’ purchases of higher calorie snacks and increase lower calorie snack sales (19). Grocery store simulations that focused solely on the promotion of healthy foods to children through marketing techniques failed to reproduce the effects seen in pricing interventions (23). More recently, Temple et al. (24) found that combining warning labels and price increases for energy drinks (ED) in a simulated corner store setting led teens to purchase fewer EDs or to swap EDs for other less expensive caffeinated products (i.e., coffee, tea, and soda). However, adults were only influenced by price increases, with decreased ED purchases being the only significant result observed (24). This suggests that – compared to adults – adolescents’ food purchasing decisions may be more strongly influenced by pricing interventions that include messaging about the health impact of a less healthy product.

Additionally, experimental studies indicate that 8- to 12-year-olds are responsive to differences in the price of snack foods. However, few retail interventions focus on influencing the relative price of foods available to children. Results from interventions that have included a price component within their intervention protocol are mixed (24, 25). An intervention at a recreational sports facility that utilized a combination of signage, taste testing and price reduction to promote healthier snack choices found no influence of price reduction on healthy snack sales. However, this may be the result of less price sensitivity in the study population due to their high socioeconomic status (25). To date, no intervention has looked at price reduction on food products in conventional retail settings despite the evidence from observational and experimental studies that pricing influences adolescent purchases.

The Coupons for Healthier Options for Minors Purchasing Snacks (CHOMPS) project is a novel intervention that applies economic, psychological, and nutritional insights to children’s autonomous food purchase behavior outside of-school. This field experiment held in the convenience store setting aimed to lead youth ages 6 to 15 away from EDNP foods and toward more healthful alternatives utilizing coupon discounts. An additional goal was to assess children’s price responsiveness related to both healthier and EDNP snack foods. This USDA-funded, community-based pilot study involved community partners and small convenience stores in Somerville, Medford, and Cambridge, Massachusetts. These stores were chosen due to their walking proximity to local schools, serving racially, ethnically, and economically diverse population.

## Materials and methods

The CHOMPS pilot was designed as a multi-disciplinary, four-part intervention involving (1) formative research, (2) natural observation, (3) in-store coupon intervention, and (4) individual assessments. The methods and results for the first three parts are reported here. The individual assessments looked at measures of cognitive, social, and language development to explore potential associations between them and youth coupon use/other purchasing habits. The study was approved by the Tufts Institutional Review Board for human subjects research.

### Formative research

The formative research portion of the study consisted of youth focus groups to develop the coupon intervention, store recruitment, and the development and use of a tool to assess snack offerings in the study stores.

#### Youth focus groups

During spring and summer 2014, the CHOMPS project conducted a series of four focus groups with youth in after-school and summer programs in Somerville, MA. Nineteen students, ages 9 to 15 years old participated in discussions about their snacking habits, shopping habits, and understanding of coupons. Coupon and poster designs were also pilot-tested. Students provided names of stores in which they and their peers shop, providing a starting point for the CHOMPS projects’ potential intervention partners.

#### Store recruitment and intervention design

Store recruitment occurred on a rolling basis from October 2014 to March 2016. Recruitment focused on stores within Somerville, Cambridge, and Medford, Massachusetts that (1) were located in racially, ethnically, and economically diverse neighborhoods; (2) were within 0.25 miles of at least one K-8 school or were mentioned during the focus groups as being along a walking route to school; and (3) carried some products that could be categorized as “healthier” snacks (see below regarding categorization process). Approximately 30 potential store partners were identified using geospatial mapping, but upon further investigation, only seven were approached to participate based on the above listed criteria. Of the seven stores approached, a total of four convenience/corner stores (which are referred to here by number only) agreed to participate in the study – two stores in Somerville (Stores 1 and 3), one in Medford (Store 2), and one in Cambridge (Store 4). The stores were dispersed geographically such that it would be very infeasible for a youth shopper to visit more than one store while walking home from school. The stores were in largely residential areas close to parks, ball fields, and some near community centers or libraries, with three of the four stores being within 0.25 miles of a school.

A Store Assessment Tool (SA) developed by the research team was then used in each store to catalog the purchasing environment, pricing, sizes, and layout of snack foods sold by store partners (copy of SA provided in Appendix). The research team developed their own assessment tool based on the Nutrition Environment Measurement Survey (NEMS)-Corner Stores (26), to gather more specific detail about snack food availability than the NEMS-CS was able to capture. The SA was programed into Qualtrics to allow research assistants to input information and photos from store visits.

Additionally, the SA was used to identify targeted (i.e., healthier) and competing (i.e., EDNP) foods suitable for discounting as a part of the pilot study. The SA categorized the healthier snack products on a two-tiered system adapted from the specified nutrient targets from the Institute of Medicine’s (IOM) (27) guidelines for competitive foods in schools (Table 1). Tier A is the optimal target for the intervention’s targeted snacks and includes fruits and vegetables, whole grain products, nonfat or low-fat dairy products, baked or low-sodium chips or pretzels, granola bars, and low-fat cookies. Additionally, based on the IOM guidelines, nuts are allowed in Tier A, even if their fat content is above the guidelines. Due to limited offerings of snacks in certain stores in the pilot (and our desire not to add new products to stores), we expanded the study guidelines to include Tier B, which allowed for slightly higher calorie and sodium content for an additional group of targeted snacks. Examples of Tier B snacks included Rice Krispy treats and animal crackers. Competing snacks were considered less healthy/unhealthy items that did not meet either the Tier A or Tier B standard. As the goal of the CHOMPS pilot was to steer children toward healthier analogs of competing snacks, the slightly higher standards for Tier B snacks still worked to support that outcome.

**Table 1:** Guidelines for targeted snacks in CHOMPS Pilot*

Tier A (nutrients in entire package)	Tier B (nutrients in entire package)
≤ 200 calories	≤300 calories
≤ 35% total calories from fat	≤ 35% total calories from fat
< 10% total calories from saturated fat	< 10% total calories from saturated fat
Zero trans-fat (≤ 0.5 g /serving trans-fat)	Zero trans fat (≤ 0.5 g /serving trans fat)
≤ 35% of calories from total sugars	≤ 35% of calories from total sugars
≤ 200 mg sodium	≤ 480 mg sodium

* Based on IOM standards for competitive foods in schools. The IOM allows nuts in the highest tier, even if their fats are over the guidelines.

### Natural observation phase

Each recruited store began the intervention with a natural observation period, which occurred on a rolling basis as each store signed on to participate in the study. Research assistants utilized the Kids Purchase Observation Tool (KPOT), an instrument developed by the research team, to record child purchases during before and after school time (copy of KPOT provided in Appendix). It was infeasible to secure parental consent to gather individual child data and/or to communicate with youth shoppers. Therefore, research assistants recorded only basic, observable data about child shoppers (estimated grade level, observed sex (male, female, and unknown), whether the child was shopping alone/with a group/with an adult), and their purchasing decisions (snack choice and total cost of purchase). The natural observation results provided baseline measurements and helped to determine which snack items were being purchased most often, forming the foundation for the schedule of items to be discounted in the coupon intervention phase.

### Coupon intervention

After the natural observation period, each store then participated in the coupon intervention, which was carried out in two phases. In Phase 1, both targeted and competing snack items were discounted and a more passive promotion strategy was used. Children only received the discounted price for a given snack item when using a coupon at the point of sale. Stores 1–3 participated in Phase 1 from October 2014 – June 2015. Phase 2 focused only on discounting targeted snack items and utilized a more active promotion strategy. Stores 2 and 4 participated in Phase 2 from September 2015 – June 2016. Ideally all stores would have continued through both phases; however, this was not possible. Store 1 closed shortly after completing Phase 1, and due to the burden on store owners of hosting the experiment, Store 3 was no longer willing to participate after Phase 1. See Table 2 for a summary of the intervention timeline.

**Table 2:** Summary of coupon intervention stages.

Natural Observation	Coupon Intervention	
Rolling start dates October 2014 – March 2016	Phase 1 October 2014 – June 2015	Phase 2 September 2015 – June 2016
All 4 stores	Stores 1, 2, and 3	Stores 2 and 4
No discounts	Discounts on targeted items and competing items.	Discounts on targeted items only.
Data collection throughout the week.	Discounts Mon-Wed and Thurs - Friday	Discounts Mon-Wed and Thurs - Friday
Natural phase with no promotions or interventions.	Passive promotional strategies (kids-only coupons and posters)	Active promotional strategies (kids-only coupons and posters, along with price messaging by RAs, health messaging by RAs, and occasional person in monkey costume)

**Figure 1:**
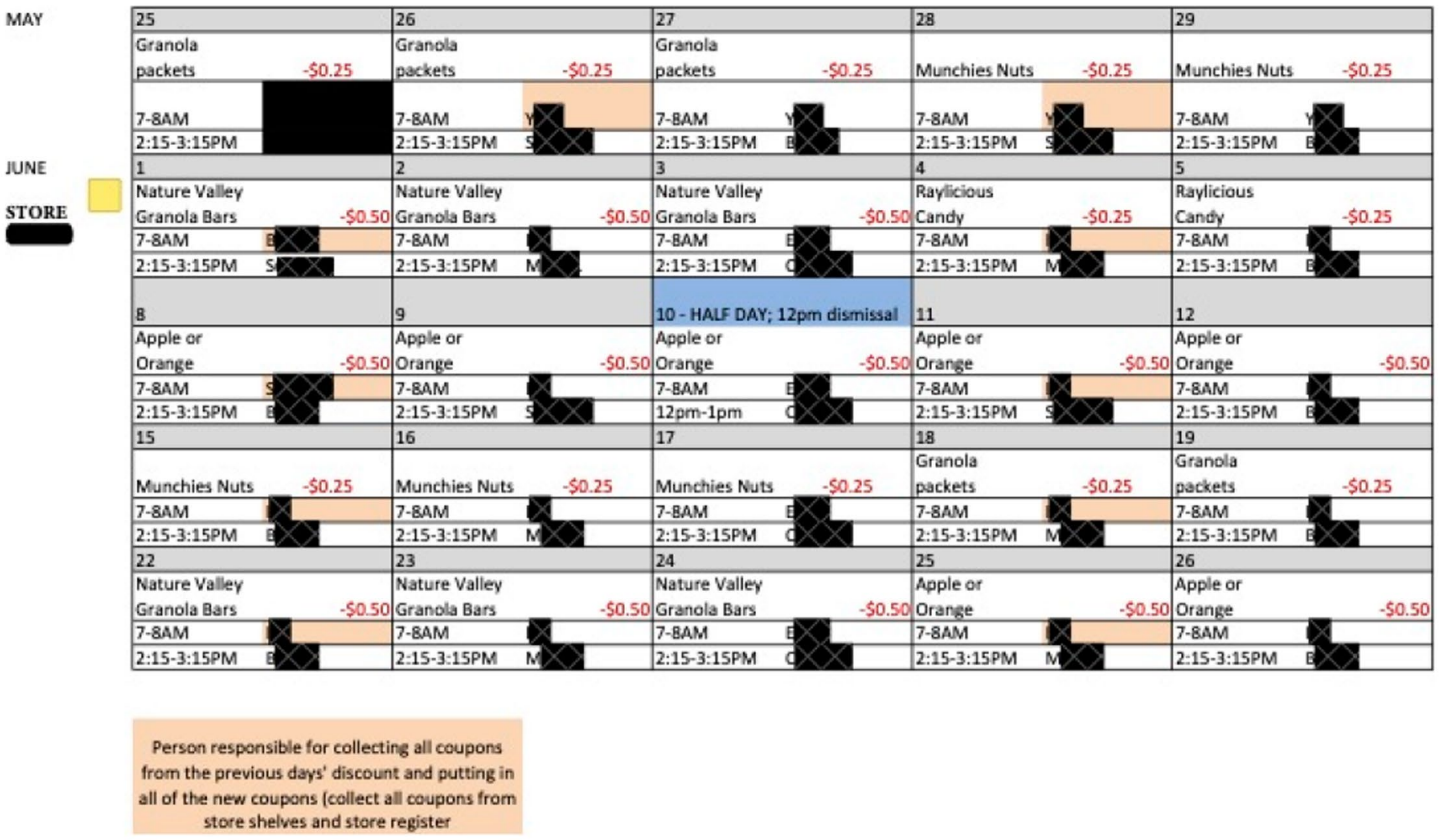
Sample discount schedule from a store in phase 1. This is a sample schedule from one month in one store only. RAs’ names have been redacted.

During each intervention phase, data from the focus groups and the natural observation phase were used to develop a weekly schedule for each store that outlined the product and discount amount that followed a Monday – Wednesday and a Thursday – Friday rotation. Coupons for the discounted item/amount for the week were placed on the shelf near the item and at the checkout counter in the store. See Figure 1 for a sample discount schedule. Discounts were offered on snacks that were sold in a package or quantity that would likely be eaten in one sitting (i.e., a one or two serving size quantity). The discount amount on the coupons was based on focus group findings and previous research by Cash and McAlister (8), which suggested $0.25 and $0.50 discounts were sufficient to be influential (8). The study piloted discount amounts of $0.10, $0.25, $0.50, and $1.00, with the majority of discounts being $0.25 or $0.50.

Research assistants were present during before and after school time to collect observational data about child purchases using the KPOT. Training was also provided for store staff to ensure that no adults were utilizing the coupons.

#### Phase 1

During Phase 1 of the coupon intervention, both targeted and competing snacks were discounted in Stores 1–3 to assess children’s price responsiveness to different snack types. Examples of targeted snacks that were discounted include: baked chips, whole fruit, nut mixes, sliced fruit or vegetables, Nature Valley granola bars, and low-fat pretzels. Examples of competing snack items that were discounted include: single-serving Doritos, Sour Patch kids, and El Isleno plantain chips. The passive promotional strategies used in this phase included posters and “kids-only coupons” signage alerting children to the presence of the coupons. The signage was designed to include animal characters and wording attractive to young shoppers, which was pilot tested during the focus groups.

#### Phase 2

During phase 2, only targeted snack items were discounted in Stores 2 and 4 and a more active promotion strategy was implemented. The [omitted] posters and signage were still present in the active promotion phase, but additionally simple, verbal messaging was used by research assistants present in the stores to encourage purchases of the targeted snack items. The messaging had either a health or economic focus, such as:

***Price Messaging.***

*“Hi! Have you seen these coupons, yet? They will save you 50 cents on ______ product!”*

***Health Messaging:***

*“Hi! Did you know you could use this coupon to save money on healthier snacks?”*

In addition to the price and health promotions, a spokesperson in a monkey costume was used intermittently, to assess any additional effect that might have on children’s purchase choices. The monkey was matched to the design of one of the CHOMPS promotional posters and was chosen because students of all ages during focus group pilot testing responded positively to the monkey character as a marketing messenger.

### Measures

Observation data from both the natural observation and coupon intervention phases in each store included information about the child shoppers and their purchasing decisions. For each purchase event, research assistants recorded the child’s estimated age range and sex, whether the child was shopping alone/with a group, whether the child paid for his/her own purchase or if another child paid, whether or not an adult was present, and whether or not an adult paid for the purchase. No other identifying information was recorded for each child; therefore, repeat shopping events over the course of the intervention for any given child were not tracked.

In addition to their demographic information, data were recorded about every item purchased regardless of package size including the brand, flavor, package size, and item price for each snack and whether or not a coupon was utilized. Because non-snack grocery items were not of interest in this study, items that were obviously grocery purchases were recorded in bulk and a total purchase cost recorded.

After all intervention phases were completed, nutrition information was linked to each of the snack purchases based on the reported brand, flavor, and package size. While drinks were not a priority for discounting in this study, the nutritional content of beverages was also included for each purchase event. Nutrition information was gathered from photos of snack packages, manufacturer websites, and the USDA Food and Nutrient Database for Dietary Studies (FNDDS), and the nutrients included for each snack item were based on the current Nutrition Facts Panel requirements: calories (kcal), total fat calories (kcal), total fat (g), calories from total fat (%), saturated fat (g), calories from sat. fat (%), trans fat (g), cholesterol (mg), sodium (mg), carbohydrates (g), fiber (g), sugar (g), calories from sugar (%), protein (g), vitamin A (%DV), vitamin C (%DV), calcium (%DV), and iron (%DV).

### Statistical analyses

Data were analyzed using Stata IC (StataCorp Statistical Software. Version 14. College Station, TX; 2017). Comparisons of purchase characteristics and nutritional content of snack purchases during the three discount periods (i.e., natural observation/no discount, targeted item discount in phases 1 and 2, and competing item discount in phase 1) were made using Chi-squared tests as well as with pairwise one-way *t*-tests comparing means between each discount period. A simple linear regression was used to assess how shopping alone versus in a group may have influenced purchasing choices. Purchase events were excluded from the analysis if they contained items that were unable to be identified. Additionally, purchases made by adults on behalf of children and purchases that included grocery items were excluded because we assumed those purchases were made with an adult’s influence/money and thus the youth was not acting autonomously.

## Results

Across all stores and intervention phases, we recorded 2,973 purchase observations totaling approximately $6,000. Out of all purchase events, the research assistants observed an almost equal split between male and female shoppers, and about 55% of purchases were made by shoppers estimated to be 10–12 years of age (Table 3). Shoppers aged 13 or older were the second most frequently observed age group, followed by those younger than nine.

**Table 3:** Shopper demographics (*n* = 2,973).

	Approximate age category (years)		
	Less than 9	10–12	13 or older
Male (%)	18.72	56.41	23.51
Female (%)	14.47	54.56	30.20

% may not equal 100 due to inability to estimate sex or age, and exclusion of purchases made by adults and those including grocery items or items unable to code.

Children were observed purchasing a range of items, but the majority of items were unhealthy snacks. Chips were most popular (38% of observed purchases), followed by candy (18%) and baked goods (12%). While the study did not involve discounting beverages, it is notable that 18% of items purchased were beverages. The most popular snack categories remained constant regardless of coupon discount type (Table 4).

**Table 4:** Item purchases as % of total by discount type (*n* = 5,892).

	Condition 0: before coupons (*n* = 2,048)^	Condition 1: targeted item discount period (*n* = 2,664)^	Condition 2: competing item discount period (*n* = 1,180)^
Chips	37.55	39.60	37.01
Candy	18.70	15.84	19.66
Drink	16.75	19.86	16.02
Packaged baked goods	14.84	8.30	15.76
Chocolate	6.64	4.32	5.93
Fruit snacks	4.00	1.95	3.05
Granola bars	0.44	0.38	0.00
Ice cream	0.15	3.60	0.93
Sandwich	0.24	1.20	0.00
Nuts and seeds	0.39	1.61	0.42
Fruit or vegetable	0.24	2.97	1.02
Other food^#^	0.05	0.38	0.17

*n* = number of items purchased during the study period indicated.

^ Column totals may not equal 100% due to rounding. The conditions correspond to the different discount periods. Condition 0 is the natural observation period prior to the introduction of coupons, which includes data from all stores. Condition 1 is when targeted items were discounted in both phase 1 and 2 and includes data from all stores. Condition 2 is when competing items were discounted in phase 1 and includes data from only stores 2 and 4.

# Other foods includes pickles, meat sticks, assorted breakfast deli items.

During both phases of the coupon intervention, only about 2.2% of purchase events included the use of a coupon for a targeted item and 3.6% for a competing item. The most popular discounted items were fresh fruit (including both sliced and whole; 20 coupons used) and Doritos (18 coupons used). When targeted products were being discounted across both study phases, children spent an average of $0.26 more per visit (*p* = 0.00), as compared to the natural observation phase when coupons had not yet been introduced (Table 5). During the natural observation phase, children were observed purchasing slightly more items per visit (2.3 items, *p* = 0.00) than during the targeted item discount periods in both phases (1.9 items) (Table 6).

**Table 5:** Purchase pattern pre- and post-coupon intervention by discount type.

	Condition 0: before coupons (*n* = 921)^	Condition 1: targeted item discount period (*n* = 1,521)^	Condition 2: competing item discount period (*n* = 531)^	*p*-values			
				*X*^2^	0 v. 1	0 v. 2	1 v. 2
Individual purchase total cost	$1.90(1.42)	$2.16(2.00)	$1.83(1.37)	0.00	0.00	0.72	0.00
Items per purchase	2.26(1.34)	1.93(1.34)	2.21(1.37)	0.86	0.00	0.79	0.00
Targeted items (for all purchase events)	0.017(0.13)	0.043(0.20)	0.02(0.15)	0.00	0.00	0.82	0.30
Total targeted items purchased~	21	107	16	0.00	0.00	0.87	0.03
Number of coupons used (% of purchases)	–	33 (2.17%)	19 (3.58%)	–	–	–	–

*n* = number of observed purchase events during the study period indicated. Data represented in mean (standard deviation) unless otherwise indicated. Excluded are purchases made by adults and those including grocery items or items were unable to be identified.

^ The conditions correspond to the different discount periods. Condition 0 is the natural observation period prior to the introduction of coupons, which includes data from all stores. Condition 1 is when targeted items were discounted in both phase 1 and 2 and includes data from all stores. Condition 2 is when competing items were discounted in phase 1 and includes data from only stores 2 and 4. Targeted items include all items that would have been eligible for discount but were not actually discounted. The total includes any instance in which targeted items were purchased during each discount period, regardless of the coupon being offered if any.

**Table 6:** Average nutrient intake for select nutrients by discount type.

	Condition 0: before coupons (*n* = 921)^	Condition 1: targeted item discount period (*n* = 1,520)^	Condition 2: competing item discount period (*n* = 531)^	*p*-values			
				*X*^2^	0 v. 1	0 v. 2	1 v. 2
Calories (kcal)	496.99 (359.40)	488.36 (405.70)	499.39 (380.85)	0.00	0.86	0.99	0.84
Calories from total fat (%)	32.34 (20.04)	28.84 (22.81)	32.18 (20.17)	0.00	0.00	0.99	0.01
Calories from sat. fat (%)	8.49 (7.29)	7.77 (8.22)	9.15 (7.91)	0.00	0.07	0.28	0.00
Calories from sugar (%)	35.78 (30.50)	38.53 (35.02)	36.08 (30.54)	0.00	0.11	0.99	0.30
Fiber (g)	2.10 (2.67)	2.49 (3.18)	2.08 (2.53)	0.00	0.00	0.99	0.01
Cholesterol (mg)	4.23 (12.61)	9.83 (47.10)	3.71 (8.38)	0.00	0.00	0.96	0.00
Sodium (mg)	503.92 (537.89)	564.02 (826.74)	477.49 (519.27)	0.02	0.10	0.77	0.40
Vitamin C (%DV)	18.40 (47.56)	28.99 (96.43)	14.65 (45.50)	0.00	0.00	0.64	0.00
Vitamin A (%DV)	3.44 (10.13)	6.62 (23.92)	2.88 (9.65)	0.00	0.00	0.84	0.00
Calcium (%DV)	8.06 (22.48)	7.31 (17.18)	6.45 (12.47)	0.00	0.59	0.24	0.62

*n* = number of observed purchase events. Results presented as mean (standard deviation) in the units indicated, unless otherwise noted. Excluded are purchases made by adults and those including grocery items or items were unable to be identified.

^ The conditions correspond to the different discount periods. Condition 0 is the natural observation period prior to the introduction of coupons, which includes data from all stores. Condition 1 is when targeted items were discounted in both phase 1 and 2 and includes data from all stores. Condition 2 is when competing items were discounted in phase 1 and includes data from only stores 2 and 4. %DV = percent Daily Value.

Based on the simple linear regression analysis, we found differences of a very small magnitude between youth shopping alone versus in a group during the targeted discount period versus the other two periods. Youth spent slightly more money when shopping alone under Condition 1 (targeted discount), bought slightly fewer items, and bought slightly more targeted items when compared to Condition 0. There were not significant differences between shopping alone vs. in a group during the competing discount period (not shown). These results essentially track with our overall study results.

Regardless of the type of discount being offered, each snack purchase event averaged 493 total calories. The percentage of calories from total fat was significantly lower during periods when targeted items were discounted (28.8%) versus when no discount was provided (32.3%; *p* = 0.00) or when competing discounts were offered (32.2%, *p* = 0.01). Additionally, the fiber (2.5 g), vitamin C (29.0 %DV), and vitamin A (23.9 %DV) content of purchases was significantly higher during periods when targeted item were discounted compared to both no discount and competing item discount periods. Lastly, the cholesterol content of snack purchases was significantly higher (9.8 mg) during targeted discount periods than non- or competing discount periods. Table 6 outlines the nutritional analysis of snack purchases.

Lastly, no significant effects were observed as a result of the use of passive (e.g., signage only) versus active (e.g., verbal cues and monkey costume) promotional techniques.

## Discussion

Based on the data presented here, initial results indicate that kids-only coupons could play a role in shifting children’s snacking behavior. Chips, candy, and drinks were found to be the most frequently purchased items. On average, children spent significantly more money and purchased slightly fewer items when healthier (targeted) items were discounted, as opposed to either no discount or an EDNP (competing) item discount. This may indicate that the coupons for targeted products allowed children to spend more during that shopping occasion on healthier snacks without the barrier of price (“price effect”), knowing that healthier options tend to be more expensive than EDNP options. Additionally, children were generally not compensating by using the coupon savings to purchase additional snack items (and may have actually been saving money). We also suspect a potential spillover effect where the coupons and messaging about healthier snack items reminded youth shoppers to purchase healthier snacks (“informational effect”). Whether they bought the snack on discount that day or not, they may have purchased fewer snacks overall during the targeted item discount period.

Additionally, a significantly higher number of targeted items were purchased during targeted item discount days. While this difference is modest in practical terms, it may indicate that the presence of coupons for healthier snacks could increase children’s interest in purchasing healthier items regardless of the specific item being discounted and/or regardless of whether a child used a coupon. When targeted coupons were present in the stores, the nutritional content of purchases were better with regard to the ten nutrients of interest to this study except for total calories, calories from saturated fat, calories from sugar, and calcium. Improvements in calories from total fat, fiber, and vitamin content may be driven by increases in fruit and vegetable snack purchases during targeted discount periods. While significantly higher cholesterol content was seen in purchases during targeted item discount periods, this could be related to higher purchases of discounted granola bars and nuts.

While no significant differences were observed between passive versus active promotional phases, a longer duration of active promotion may be necessary to observe effects. Additionally, it is likely that many observed children were repeat customers in a given study store. If they were exposed first to the passive promotion strategies, they may not have reacted substantially to the shift to more active promotion.

## Strengths and limitations

This pilot study is the first of its kind to assess the feasibility and potential impact of healthier snack coupons meant for autonomous youth purchasers. Major study design strengths include the real-world setting and non-invasive observational approach that should have minimized any observer effects. Despite the non-invasive approach, researchers tracked a full or nearly full set of children’s autonomous purchases in order to capture spillover effects, including increased purchases of healthier items that were not being discounted. An additional strength was the use of the IOM’s guidelines for competitive foods in schools to determine discountable items.

This study is not without limitations. Most importantly, we were unable to record individualized child data including demographics and repeat purchases, since it would have been infeasible to acquire parental consent for these young shoppers observed in actual retail settings. Therefore, we could not analyze individual-level data (beyond apparent age and sex) to infer who was buying which products and why and/or control for confounders like income level. This limitation is the flipside of the strength of the observational approach, and it is possible that individualize data could reveal a smaller effect from the coupons and/or effects only with certain demographic groups. This pilot study also occurred only in a handful of inner suburbs of Boston in 2014–2016. While neither of these should impact the internal validity of the results and in general we do not anticipate youth’s autonomous purchasing behavior to have shifted much in that time, it is possible that the same results would not be observed today or be readily generalizable to other locations.

Additionally, the Store Assessment Tool and KPOT used in the study were designed for use in this specific study and did not go through separate validity testing. The validated NEMS-CS^26^ instrument did provide the foundation for our Store Assessment tool; however, additional components were added to gather more granular data about healthy and less healthy snack availability and pricing. While thorough training was provided to enumerators to ensure reliable data collection, the Store Assessment and KPOT should go through separate validity testing if to be used in a future trial.

Lastly, the data collection methods used were extremely time intensive. The researchers sought to work with small community partners to design a replicable intervention; however, there may be self-selection bias at play with regards to the stores who opted into the intervention. Working with larger stores and/or chains may bring efficiency of scale and allow for technology, like electronic point-of-sale systems, to streamline data collection.

## Contribution to the field

Child nutrition interventions often focus on schools or home settings and aim to improve health-related behaviors both inside and outside of those environments. While it can be challenging to measure the spill-over effects of these types of interventions, some evidence suggests that children’s autonomous snack purchases will increase in community settings if snack availability is limited in the household or school (20, 28). Anecdotes from this study support this scenario, in that researchers sometimes overheard statements that children were buying large bags of chips and liter sodas because they were not going to have access to them at home over the weekend. Therefore, more research must aim to influence children as autonomous purchasers in grocery or convenience store settings. Results from this pilot indicate that kids-only coupons have the potential to assist with shifting snacking behavior outside of school settings, specifically when kids are using their own money to make purchase decisions. These are promising results that provide an effect size estimate that could be used to design a fully powered randomized control trial. We anticipate that such an intervention would be even more impactful when combined with healthy snacking marketing campaigns and interventions focused on creating healthier corner stores – a strategy that is increasing in prominence (29–31).

## Data availability statement

The raw data supporting the conclusions of this article will be made available by the authors, without undue reservation.

## Ethics statement

The studies involving humans were approved by Tufts University Social Behavioral and Educational Research Institutional Review Board (SBER-IRB). The studies were conducted in accordance with the local legislation and institutional requirements. The ethics committee/institutional review board waived the requirement of written informed consent for participation from the participants or the participants’ legal guardians/next of kin because purely observational study with no data collection involving direct interaction with children.

## Author contributions

MM: Conceptualization, Data curation, Formal analysis, Investigation, Methodology, Project administration, Software, Visualization, Writing – original draft, Writing – review & editing. AM: Conceptualization, Investigation, Methodology, Writing – review & editing. CE: Conceptualization, Investigation, Writing – review & editing. SM: Data curation, Project administration, Writing – review & editing. KP: Data curation, Project administration, Writing – review & editing. SC: Conceptualization, Formal analysis, Funding acquisition, Investigation, Methodology, Project administration, Supervision, Validation, Writing – review & editing.

## References

[1] Borradaile KE Sherman S Vander Veur SS McCoy T Sandoval B Nachmani J. Snacking in children: the role of urban corner stores. Pediatrics. (2009) 124:1293–8. doi: 10.1542/peds.2009-0964, PMID: 19822591

[2] Dennisuk LA Coutinho AJ Suratkar S Surkan PJ Christiansen K Riley M. Food expenditures and food purchasing among low-income, urban, African-American youth. Am J Prev Med. (2011) 40:625–8. doi: 10.1016/j.amepre.2011.02.015 21565654

[3] Jones S Chu YH Burke MP Teixeira A Blake CE Frongillo EA. A case for targeting marketing and availability in school food policy: Adolescents' food purchases at school and exposure to television, internet, and video games. J Hung Environ Nutr. (2012) 7:1–10. doi: 10.1080/19320248.2012.651389

[4] Lent MR Veur SV Mallya G McCoy TA Sanders TA Colby L. Corner store purchases made by adults, adolescents and children: items, nutritional characteristics and amount spent. Public Health Nutr. (2015) 18:1706–12. doi: 10.1017/S1368980014001670, PMID: 25115817 PMC4720486

[5] Sherman S Grode G McCoy T Vander Veur SS Wojtanowski A Sandoval BA. Corner stores: the perspective of urban youth. J Acad Nutr Diet. (2015) 115:242–8. doi: 10.1016/j.jand.2014.10.017, PMID: 25636219

[6] Marshall D. Convenience stores and discretionary food consumption among young Tokyo consumers. Int J Retail Distrib Manag. (2016) 44:1013–29. doi: 10.1108/IJRDM-08-2015-0137

[7] Engler-Stringer R Schaefer J Ridalls T. An examination of the roles played by early adolescent children in interactions with their local food environment. Can J Public Health. (2016) 107:eS48–52. doi: 10.17269/CJPH.107.5296 PMC6972376 27281514

[8] Cash SB McAlister A.R. Influence of developmental differences on Children's response to information on foods. United States Department of Agriculture (2011). doi: 10.7910/DVN/UTXFBP

[9] Setiono FJ Gangrade N Leak TM. U.S. adolescents’ diet consumption patterns differ between grocery and convenience stores: National Health and nutrition examination survey 2011–2018. Int J Environ Res Public Health. (2021) 18:8474. doi: 10.3390/ijerph18168474 34444223 PMC8394683

[10] Vander Veur SS Sherman SB Lent MR McCoy TA Wojtanowski AC Sandoval BA. Corner store and commuting patterns of low-income, urban elementary school students. Curr Urban Stud. (2013) 1:166–70. doi: 10.4236/cus.2013.14018

[11] Signorielli N Lears M. Television and Children's conceptions of nutrition: unhealthy messages. Health Commun. (1992) 4:245–57. doi: 10.1207/s15327027hc0404_1

[12] Farrell L Shields MA. Children as consumers: investigating child diary expenditure data. Can J Econ Revue Canadienne D Economique. (2007) 40:445–67. doi: 10.1111/j.1365-2966.2007.00416.x

[13] Lundberg S Romich JL Tsang KP. Decision-making by children. Rev Econ Househ. (2009) 7:1–30. doi: 10.1007/s11150-008-9045-2

[14] Gangrade N Figueroa J Leak TM. Socioeconomic disparities in foods/beverages and nutrients consumed by U.S. adolescents when snacking: National Health and nutrition examination survey 2005–2018. Nutrients. (2021) 13:2530. doi: 10.3390/nu13082530 34444690 PMC8399168

[15] Epstein LH Handley EA Dearing KK Cho DD Roemmich JN Paluch RA. Purchases of food in youth - influence of price and income. Psychol Sci. (2006) 17:82–9. doi: 10.1111/j.1467-9280.2005.01668.x 16371148

[16] Jensen JD Bere E de Bourdeaudhuij I Jan N Maes L Manios Y. Micro-level economic factors and incentives in Children's energy balance related behaviours findings from the ENERGY European cross-section questionnaire survey. Int J Behav Nutr Phys Act. (2012) 9:136. doi: 10.1186/1479-5868-9-136, PMID: 23171289 PMC3514146

[17] He M Tucker P Gilliland J Irwin JD Larsen K Hess P. The influence of local food environments on Adolescents' food purchasing behaviors. Int J Environ Res Public Health. (2012) 9:1458–71. doi: 10.3390/ijerph9041458, PMID: 22690205 PMC3366623

[18] Hearst MO Pasch KE Laska MN. Urban v. suburban perceptions of the neighbourhood food environment as correlates of adolescent food purchasing. Public Health Nutr. (2012) 15:299–06. doi: 10.1017/S1368980011002114, PMID: 21859510 PMC3461946

[19] Salvy S-J Kluczynski MA Nitecki LA O'Connor BC. Peer influence on youth's snack purchases: a laboratory analog of convenience store shopping. Eat Behav. (2012) 13:233–9. doi: 10.1016/j.eatbeh.2012.03.005, PMID: 22664402 PMC7213050

[20] van Ansem WJC Schrijvers CTM Rodenburg G van de Mheen D. Children's snack consumption: role of parents, peers and child snack-purchasing behaviour. Results from the INPACT study. Eur J Pub Health. (2015) 25:1006–11. doi: 10.1093/eurpub/ckv098, PMID: 26045526

[21] Caraher M Lloyd S Mansfield M Alp C Brewster Z Gresham J. Secondary school pupils' food choices around schools in a London borough: fast food and walls of crisps. Appetite. (2016) 103:208–20. doi: 10.1016/j.appet.2016.04.016 27105582

[22] Elbel B Tamura K McDermott ZT Duncan DT Athens JK Wu E. Disparities in food access around homes and schools for new York City children. PLoS One. (2019) 14:e0217341. doi: 10.1371/journal.pone.0217341, PMID: 31188866 PMC6561543

[23] Heard AM Harris JL Liu S Schwartz MB Li X. Piloting an online grocery store simulation to assess children's food choices. Appetite. (2016) 96:260–7. doi: 10.1016/j.appet.2015.09.020 26409642

[24] Temple JL Ziegler AM Epstein LH. Influence of Price and labeling on energy drink purchasing in an experimental convenience store. J Nutr Educ Behav. (2016) 48:54–59.e1. doi: 10.1016/j.jneb.2015.08.007 26404774

[25] Olstad DL Goonewardene LA McCargar LJ Raine KD. Choosing healthier foods in recreational sports settings: a mixed methods investigation of the impact of nudging and an economic incentive. Int J Behav Nutr Phys Act. (2014) 11:6. doi: 10.1186/1479-5868-11-6, PMID: 24450763 PMC3901328

[26] Cavanaugh E Mallya G Brensinger C Tierney A Glanz K. Nutrition environments in corner stores in Philadelphia. Prev Med. (2013) 56:149–51. doi: 10.1016/j.ypmed.2012.12.007, PMID: 23262362

[27] Medicine Io. Nutrition standards for foods in schools: Leading the way toward healthier youth. Washington, DC: The National Academies Press (2007).

[28] Downs S Demmler KM. Food environment interventions targeting children and adolescents: a scoping review. Glob Food Sec. (2020) 27:100403. doi: 10.1016/j.gfs.2020.100403

[29] Winkler MR Lenk KM Erickson DJ Caspi CE Laska MN. Longitudinal fruit and vegetable sales in small food retailers: response to a novel local food policy and variation by neighborhood socioeconomic status. Int J Environ Res Public Health. (2020) 17:5480. doi: 10.3390/ijerph17155480 32751326 PMC7432731

[30] Karpyn A DeWeese RS Pelletier JE Laska MN Ohri-Vachaspati P Deahl-Greenlaw A. Examining the feasibility of healthy minimum stocking standards for small food stores. J Acad Nutr Diet. (2018) 118:1655–63. doi: 10.1016/j.jand.2017.12.006 29650459

[31] Laska MN Sindberg LS Ayala GX D’Angelo H Horton LA Ribisl KM. Agreements between small food store retailers and their suppliers: incentivizing unhealthy foods and beverages in four urban settings. Food Policy. (2018) 79:324–30. doi: 10.1016/j.foodpol.2018.03.001

